# Toward resilient and efficient pediatric critical care provision amid falling birth rates

**DOI:** 10.1007/s12519-026-01087-6

**Published:** 2026-09-15

**Authors:** Gang Liu, Quan Wang, Su-Yun Qian

**Affiliations:** Pediatric Intensive Care Unit, Beijing Children’s Hospital, Capital Medical University, National Center for Children’s Health; National Clinical Research Center for Respiratory Diseases, Beijing 100045, China

The global trend of declining birth rates is becoming more pronounced [1]. In China, the number of annual births has decreased from 2018 to 2025 (Fig. 1), falling well below the population replacement level [2]. Against this demographic backdrop, some medical institutions in China have closed or downsized their pediatric departments due to this shrinking patient base. This trend has also impacted the subspeciality of pediatric intensive care and, therefore, their specific units (PICUs). Moreover, Japan and South Korea, as representative nations with low birth rates, have both experienced a sustained decline in pediatric outpatient visits [3, 4]. A case study of three Chinese children's hospitals at different tiers between 2023 and 2025 reveals a decline in patient volume and the proportion of infants < 1 year, alongside a rise in median age (Table 1). These shifts reflects the impact of declining neonatal births on the age distribution of children admitted to the PICU.

**Fig. 1:**
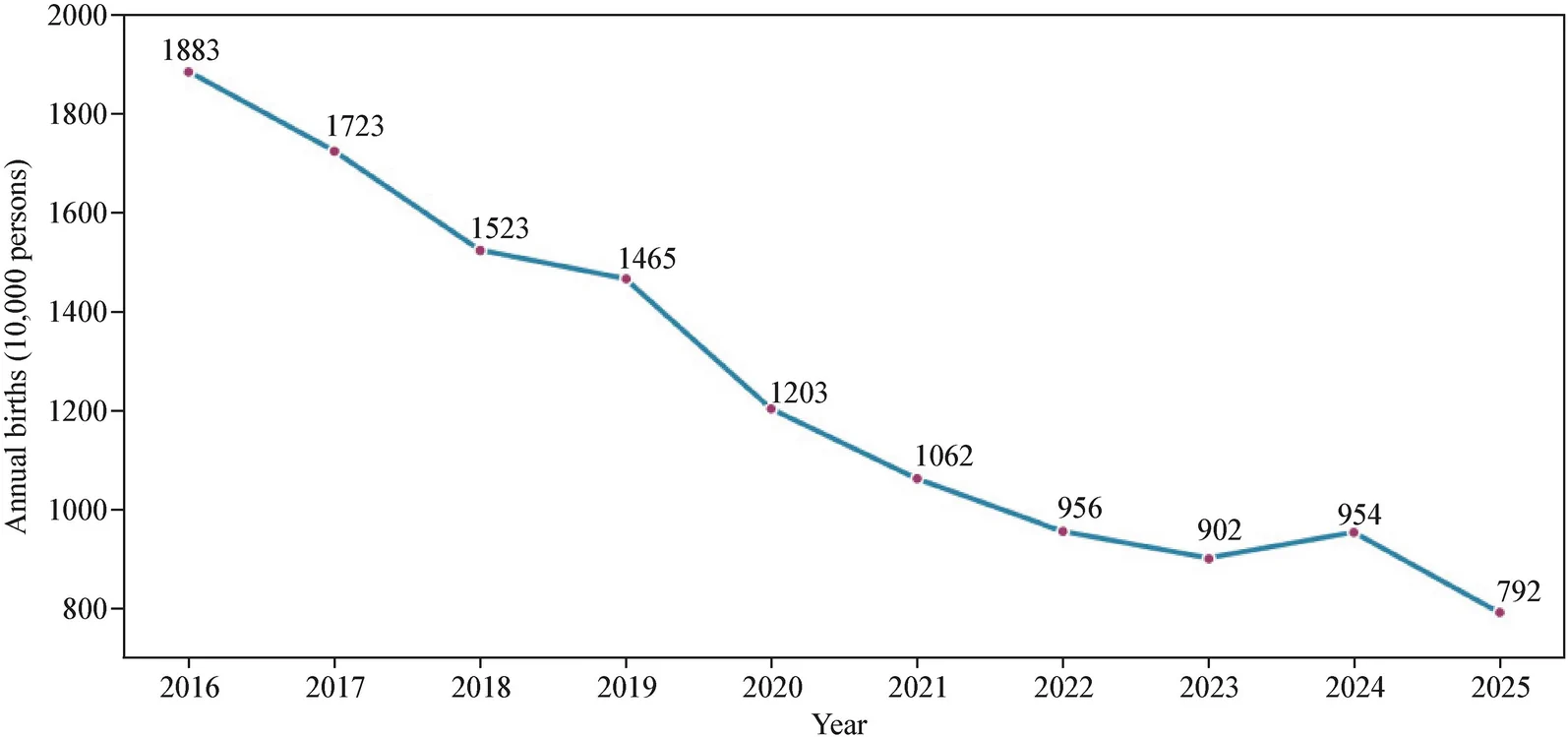
Birth trends in China, 2016–2025

**Table 1:** Trends in patient composition in three Chinese children’s hospitals by tier, 2023–2025

Year	National level			Provincial level			Municipal level		
	Patient volume	Age (mon), quartile	Proportion aged < 1 y, %	Patient volume	Age (mon), quartile	Proportion aged < 1 y, %	Patient volume	Age (mon), quartile	Proportion aged < 1 y, %
2023	1237	67 (21, 123)	18.5	602	36 (10, 96)	26.7	1619	36 (14, 84)	25.2
2024	1270	81 (22, 126)	17.9	603	48 (11, 108)	25.2	1097	40 (12, 89)	25.7
2025	1091	82 (26, 139)	15.5	582	47 (13, 108)	23.7	1096	60 (24, 108)	22.5

Historically, the development of pediatric critical care medicine in China has been underpinned by a "scale-driven" logic, where expanding service volume was key to reducing costs, sustaining operations, and training personnel. Recent high-impact studies have highlighted imbalances in pediatric healthcare resource allocation across multiple economies [5–10]. This raises a critical question: will the declining birth rate in China trigger a similar cycle of adverse development in pediatric critical care (Fig. 2), leading to a regression in the equity, rationality, and fairness of medical resource distribution? In response to this question, we analyze the current challenges and opportunities within the pediatric critical care sector. We propose a new development philosophy centered on "resilience" and "efficiency", moving beyond the traditional "scale-driven" model. This framework aims to provide strategic recommendations for pediatric critical care to navigate future challenges.

**Fig. 2:**
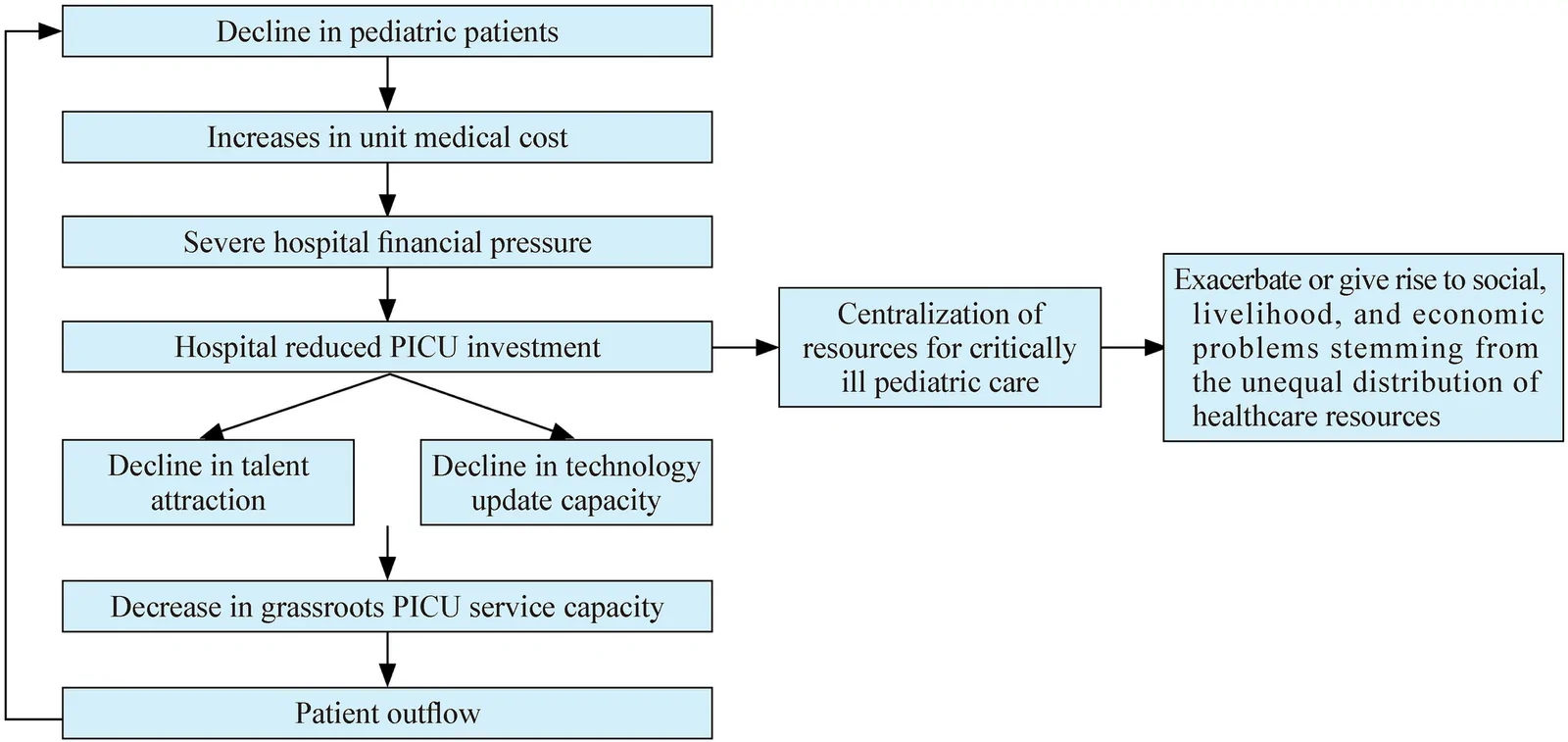
Potential unfavorable development trajectories in pediatric critical care. *PICU* pediatric intensive care unit

Rather than producing a systematic review, we utilized purposive citation. Cases were selected based on typicality (representative cases in the field of pediatric), timeliness (post-2020), and relevance to the central argument (selected cases should illustrate how PICUs can achieve sustainable development through enhanced resilience and efficiency, amid declining fertility).

## Challenges and opportunities in pediatric critical care amid declining birth rates

### Challenges: current realities

#### Instability of the workforce

The training and retention of pediatric intensivists is a prolonged process characterized by high professional demands and intense workloads. Coupled with relatively low compensation and limited career appeal, this has resulted in a global shortage of high-level talent in the field [11, 12]. Studies indicate that among 146 ICUs globally admitting pediatric patients, only 52.7% have pediatric intensivists available around the clock [5]. In China, the attrition rate among pediatricians reached 10.7% between 2011 and 2014 [13].

#### Declining patient volumes exert significant financial strain, jeopardizing the sustainability of PICU resources

For instance, during the COVID-19 pandemic, rural township hospitals in two Chinese counties witnessed a 40%–50% drop in combined outpatient and inpatient visits; this was accompanied by a 50% reduction in annual revenue [14]. The pronounced financial strain currently facing Chinese pediatrics [15] inevitably jeopardizes the sustainability of PICU investments and institutional viability. Data from 983 U.S. emergency departments indicate that the unit operating cost in low-volume pediatric departments is over 70 times higher than in high-volume settings [16]. Furthermore, insufficient investment in resources has consequences; between 2010 and 2022, only 37% of Japanese children requiring mechanical ventilation were admitted to an ICU environment due to resource constraints [7].

#### Imperfect triage mechanisms and issues of equity

Scientifically driven hierarchical diagnosis and treatment mechanisms remain incomplete, hindering healthcare equity. The United States employs a market-based, mandatory insurance system; however, this faces criticism for coverage gaps and poor primary care accessibility [17]. Between 2008 and 2018, approximately one-fifth of U.S. hospitals closed their pediatric departments, significantly reducing services in rural and low-volume urban areas [9, 18] and compromising care quality for complex cases in these regions [19]. China advocates for a non-mandatory model featuring initial primary care consultation and bidirectional referrals [20]. While aiming for resource equilibrium, this system still struggles with the "siphoning" effect, where top-tier hospitals attract a disproportionate share of patients, leaving primary care capabilities as a significant bottleneck [21].

### Opportunities

#### Policy support

Many countries are increasing policy support. The United States and South Korea, for example, are addressing the common challenge of ensuring adequate pediatrician income by raising service fees and diversifying revenue streams [22, 23]. China has also implemented a series of supportive measures, including salary incentives, optimization of service networks, and adjustments to medical insurance pricing [24, 25]. These initiatives lay a crucial economic foundation for the sustainable development of pediatric critical care medicine, though their full and effective implementation still faces obstacles.

#### Advances in treating major diseases

The increasing complexity of pediatric diseases is driving upgrades in diagnostic and treatment capabilities. In China, for instance, the mortality rate for pediatric acute necrotizing encephalopathy has dropped from 50.0% to 16.7% [26], and for septic shock from 47.4% to 23.4% [27, 28]. These improvements are attributed to knowledge updates, optimized treatment strategies, and enhanced overall service capacity in the field.

#### Artificial intelligence empowerment

The global proliferation of application-level medical artificial intelligence (AI) is beginning to play a role in disease management and prognosis improvements. Indeed, AI is assisting in reducing mortality from sepsis and shortening hospital stays [29], lowering the incidence of several perinatal maternal and infant diseases [30], and enabling personalized pain management for children [31]. However, medical AI still faces challenges, such as inconsistent data quality, lack of algorithmic interpretability, and high clinical implementation costs. This requires precise adaptation to clinical scenarios rather than indiscriminate application. Nevertheless, with appropriate regulation, AI remains an excellent method for enhancing healthcare capabilities.

#### Collaborative networks

Collaborative networks are promoting PICU tiering and integrated data research. Germany has established a pediatric surveillance network that covers nearly all children's hospitals, facilitating nationwide observational studies and providing a basis for PICU functional tiering and certification [32]. China is undertaking similar efforts based on hospital alliances, including the development of PICU quality control standards, the construction of large-sample databases, and broader multicenter cohort studies.

#### Frontier therapies

Emerging technologies, such as brain-computer interfaces, cell therapy, and gene editing, are increasingly being explored in adult medicine, offering new hope for many intractable pediatric diseases. However, their application in children faces hurdles, including insufficient evidence-based data, low technological accessibility, and incomplete ethical guidelines. Therefore, whilst these are encouraging innovations, their safe translation into clinical practice requires more world. 

#### Integration into a "life-course" health system

Both the World Health Organization and the Chinese government advocate for a "life-course" approach to health [33, 34]. Italian experience demonstrates that integrating primary care networks based on communities, supported by tertiary pediatric centers, optimizes resource allocation and enhances service continuity [35, 36]. Singapore has developed a full-chain, hospital to community model for populations in need of perinatal and pediatric palliative care [37]. Pediatric critical care can draw on these concepts to expand extended services, including outpatient, home-based, and telemedicine care [38, 39], to reduce the burden of hospitalization and improve the patient experience.

## The "resilience and efficacy" conceptual framework

"Resilience" is the core capacity to withstand shocks, such as fluctuations in patient volume, financial pressure, and talent attrition. It encompasses three dimensions: human resources (stability and long-term growth), financial (offsetting revenue gaps via subsidies, cost control, and service enhancement), and clinical (advancing treatment for complex cases, prioritizing quality over quantity). "Efficacy" refers to the transformation of opportunities into new capabilities, comprising technology (AI, telemedicine, translational research), collaboration (data sharing and partnerships), and value (improved cure rates and life-course care) (Table 2). Resilience and efficacy are mutually dependent and reinforcing.

**Table 2:** Conceptual framework for resilience and efficiency

Core concept	Dimension	Key indicators
Resilience	Talent resilience	Low physician turnover; well-established training system
	Financial resilience	Timely disbursement of policy subsidies; controllable unit costs
	Clinical resilience	Capacity to manage complex and intractable diseases; capability to respond to public health emergencies
Efficiency	Technical efficiency	Coverage of artificial intelligence-assisted diagnosis; utilization of telemedicine
	Collaborative efficiency	Level of data sharing; output of multi-center research
	Value efficiency	Long-term quality of life among pediatric patients; family satisfaction level
Expected outcomes	Maintain pediatric intensive care unit (PICU) service capacity commensurate with local population needs. Under a tiered diagnosis and treatment system: (1) avoid siphoning of low-acuity patients by central healthcare institutions; (2) ensure stable staffing and resource inputs across all PICUs; (3) prevent waste of medical resources; (4) maintain sound financial status; (5) establish a well-coordinated development system for social medical institutions; (6) develop a standardized protocol for identifying, managing, and referring critically ill children; (7) prioritize early intervention and promote early adoption of new technologies with support from tertiary hospitals	

## Toward "resilience and efficacy": core pathways and future actions

### Providing sustained technical and capacity support for tiered diagnosis and treatment

**Fig. 3:**
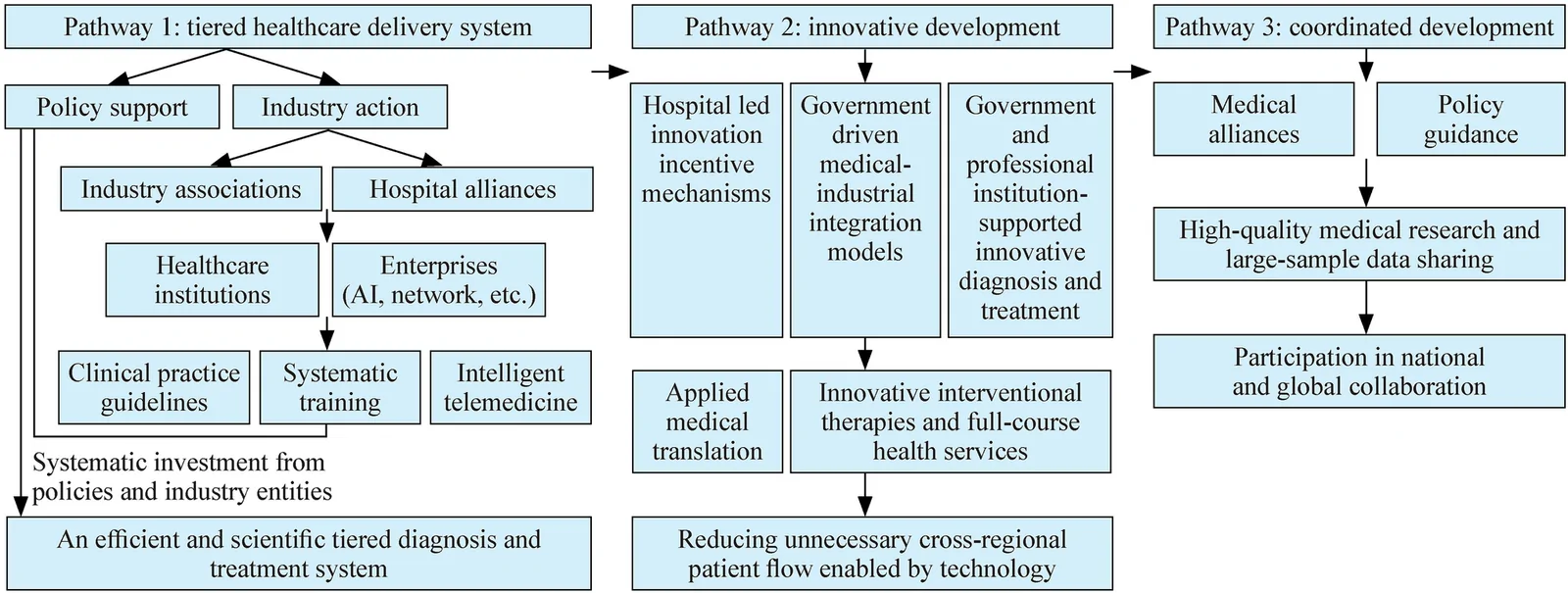
Implementation pathways using the concept of "resilience and efficiency". *AI* artificial intelligence

A tiered system is a prerequisite for rational allocation of patient resources, continuous improvement of primary care capabilities, effective utilization of talent, and realization of a "life-course" health management vision for children. Global experience indicates that the successful implementation of tiered diagnosis and treatment is closely linked to strong policy guidance and medical insurance payment reforms [40, 41]. Governments should increase investment, while the healthcare sector should eliminate technical barriers by establishing clinical standards, organizing skills training, and collaborating with technology enterprises to advance telemedicine and smart referral systems (Fig. 3, Pathway 1).

### Innovation serves as the pivotal enabler for achieving a resilient and efficient healthcare system

Technological empowerment forms the foundation for collaborative efficacy. The "Baichuan" pediatric AI model, developed under the leadership of Beijing Children's Hospital, has demonstrated a diagnostic accuracy of 82% [42] and has been deployed in over 150 hospitals across China. Concurrently, a South Korean research team developed a digital platform for "optimal hospital referral", which reduced patient mortality rates [43]. These initiatives demonstrate that AI and digital tools can effectively dismantle experiential barriers in primary care and enhance diagnostic, treatment, and referral efficiency. This pathway necessitates high-level, deep collaboration among medical institutions, research institutes, and enterprises, guided by governmental policy (Fig. 3, Pathway 2).

### Data integration is the foundation of synergistic efficacy

Institutions, such as Beijing Children’s Hospital and Fudan University Children’s Hospital, have published pivotal research based on large-scale data in top-tier journals, such as *The Lancet* and *JAMA*, addressing critical medical and social issues in oncology and congenital heart disease [44, 45]. However, systemic barriers persist, including difficulties in data sharing, lack of standardized protocols, and a lack of incentives. To address this, government-led initiatives must establish unified standards and incentives to foster inter-institutional collaboration and global database construction, thereby supporting tiered diagnosis and treatment and optimized resource allocation (Fig. 3, Pathway 3).

## Summary

Based on observations from the PICU field and limited data, this paper proposes that PICUs should shift towards a development paradigm of "resilience" and "efficacy" amidst declining birth rates. This study has several limitations. First, core arguments lack robust evidence and financial modeling. Second, proposed pathways are limited by a lack of interdisciplinary integration (spanning operations, governance, and economics). Third, we did not address acceptance within the PICU community, risk assessment, specific talent development strategies, or selection bias in the literature. Consequently, this paper offers a conceptual framework and preliminary roadmap rather than a comprehensive solution. We welcome further discussion.

## Data Availability

Data sharing is not applicable as no new data were created or analyzed
